# Cardiovascular Risk and Its Presentation in Chronic Kidney Disease

**DOI:** 10.3390/jcm14134567

**Published:** 2025-06-27

**Authors:** Stefan J. Schunk, Paul Zimmermann

**Affiliations:** 1 Department of Internal Medicine IV, Nephrology and Hypertension, Saarland University, 66421 Homburg/Saar, Germany; schunkstefan@gmx.de; 2 Department of Nephrology, Hypertension and Rheumatic Disease, Klinikum Bamberg, 96049 Bamberg, Germany; 3 Department of Cardiology, Klinikum Bamberg, 96049 Bamberg, Germany; 4 Division of Exercise Physiology and Metabolism, BaySpo-Bayreuth Center of Sport Science, University of Bayreuth, 95440 Bayreuth, Germany; 5 Interdisciplinary Center of Sportsmedicine Bamberg, Klinikum Bamberg, 96049 Bamberg, Germany

**Keywords:** chronic kidney disease, cardiovascular risk, cardiovascular biomarkers, coronary arterial disease, inflammation, renin–angiotensin–aldosterone system

## Abstract

**Background/Objectives**: Patients with chronic kidney disease (CKD) are associated with a significantly elevated cardiovascular risk. The incidence and prevalence of mediated cardiac disorders and major adverse cardiac events (MACEs), such as heart failure, arrhythmias, acute coronary syndrome (ACS) based on coronary artery disease (CAD), stroke, venous thromboembolism, and peripheral artery disease, are significantly higher in CKD patients as compared with the general population. **Methods**: This narrative review summarizes the current clinical understanding, the pathophysiological mechanisms, and the clinical consequences in the context of cardiovascular risk and disease in CKD. **Results**: The impact of CKD on mediated cardiovascular disorders and elevated MACE prevalence is complex and multifactorial. The underlying mechanisms involve various traditional cardiovascular risk factors, such as arterial hypertension, smoking, dyslipidemia, and diabetes. Furthermore, CKD-specific molecular and pathophysiological factors, such as chronic inflammation and associated oxidative stress and endothelial cell dysfunction, pro-coagulatory status, uremic toxins and uremic lipids, progressive vascular calcification, and alterations in the regulation of the renin–angiotensin–aldosterone system (RAAS) and sympathetic activation cause an increased cardiovascular risk. **Conclusions**: Understanding the complex disease mechanisms between CKD and elevated cardiovascular risk might contribute to optimizing individual patients’ risk stratification and result in individualized diagnostic and treatment strategies via appropriate clinical biomarker application and individualized anti-inflammatory approaches.

## 1. Introduction

**Figure 1:**
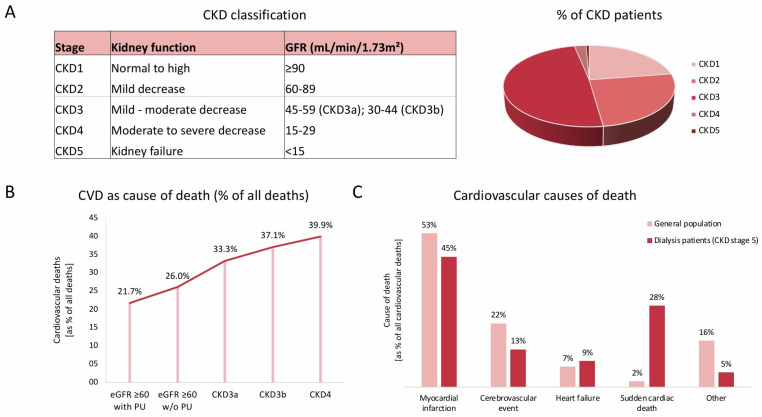
Chronic kidney disease (CKD) and associated elevated cardiovascular risk, adapted from [2]. (**A**) CKD classification; (**B**) CKD and cardiovascular death (CVD); (**C**) CVD in general population and CKD.

Chronic kidney disease (CKD) increases the risk of various associated adverse outcomes during a patient’s lifetime, whereby the increased risk and coincidence of different subtypes of cardiovascular disease (CVD) are a well-studied and recognized topic in the scientific community [1,2]. Worldwide, 15–20% of adults are affected by impaired renal function, as defined by a low glomerular filtration rate or high albuminuria [1]. In this context, CKD patients display an increased risk for various associated adverse outcomes, whereby CVD represents the most important and prognostic manifestation [1,2]. While the coincidence of CVD and individuals with healthy kidney function is estimated to be 37.5%, the coincidence of CVD and severely impaired kidney function (CKD stage 4) shows a significant increase up to 75.3% in this high-risk population [1]. Therefore, CKD is recognized as an independent risk factor for CVD by increasing cardiovascular morbidity and mortality even after optimization and individual adjustment of traditional cardiovascular risk factors [1,3]. Recent research has revealed the potential prognostic implications for assessing creatine-based estimated glomerular filtration rate (eGFR) and albuminuria for the prediction of cardiovascular outcomes [3]. The current CKD staging, its clinical prevalence, and associations with major adverse cardiac events (MACEs) are displayed in Figure 1. CKD is associated with various negative CVD outcomes, whereby the term MACEs has to be used cautiously due to its heterogeneity in the literature, mainly covering heart failure, arrhythmias, acute coronary syndrome (ACS) based on coronary artery disease (CAD), ischemic or hemorrhagic stroke, venous thromboembolism, and peripheral artery disease, as displayed in Figure 1 [3,4,5,6,7]. CKD is a known independent risk factor for CAD, even after adjustment for traditional risk factors, such as accompanying arterial hypertension and diabetes [7]. A decline in eGFR to below 60–75 mL/min/1.73 m^2^ significantly increases the clinical probability of CAD, while individuals with CKD stages G3a to G4 (15–60 mL/min/1.73 m^2^) show two to three times higher CVD mortality risk [7,8]. The underlying pathophysiological mechanisms are complex and multifactorial. Along with the impact and the role of the established classical cardiovascular risk factors, such as arterial hypertension, diabetes, dyslipidemia, and smoking, CKD also displays several specific pathophysiological and molecular mechanisms aggravating cardiovascular risk [2]. In this complex interplay, several mechanisms, such as oxidative stress, endothelial dysfunction, inflammation, and subsequent vascular calcification, as well as uremic toxins, anemia, and the overwhelming activation of the renin–angiotensin–aldosterone system (RAAS) and sympathetic activation, seem to play an important role accompanying increased CVD, as highlighted in the literature [9,10,11,12,13,14,15,16,17,18,19,20,21,22]. Due to this complex interplay, uremic cardiomyopathy is a recognized and multifactorial disease causing significant morbidity and mortality in end-stage kidney disease patients through the development of arrhythmias, heart failure, and sudden cardiac death [23].

The present narrative review aimed to describe the CKD-specific pathophysiological and molecular mechanisms along with the established classical cardiovascular risk factors and their potential impact on CVD in CKD individuals. Therefore, we want to focus on some general treatment strategies and provide recommendations to reduce cardiovascular risk in CKD patients, as well as raising general awareness regarding the complex pathophysiological mechanisms in CKD patients and highlighting potential therapeutic approaches. Additionally, our review aimed to examine the therapeutic implications associated with the use of rational biomarker assessment in the clinical routine.

## 2. Cardiovascular Risk and Its Presentation in CKD

Previous research revealed an elevated cardiovascular risk for MACEs in patients with CKD [2,24] (Figure 1). In this context, cardiovascular risk is significantly elevated as compared to the general population, even in patients with early-stage CKD (CKD stages 1–3) [24]. Patients with worsening CKD—represented by advanced CKD stages (CKD stages 4–5)—exhibit a markedly elevated risk [24]. As an underlying pathology, arteriosclerosis is estimated to represent a key process contributing to cardiovascular injury and increased cardiovascular risk in CKD [24,25].

Due to this complex interplay, i.e., the associated systemic pro-inflammatory state and subsequent myocardial and vascular remodeling, myocardial fibrosis, and vascular calcification, uremic cardiomyopathy is a recognized disease causing significant morbidity and mortality in end-stage kidney disease patients [2,23,24,25,26,27]. In this context, two major mechanisms are considered to play an essential role in the development of CVD in CKD: firstly, chronic kidney injury and damage are accompanied by the release of cytokines, hormones, and enzymes which are associated with characteristic long-term changes in the vasculature [28,29,30,31,32,33]. Secondly, cardiac damage is mediated by CKD-associated mediators with subsequent functional and morphological hemodynamic alterations [24,34]. 

The bidirectional relationship between CKD and cardiovascular dysfunction is acknowledged, but not deeply explored. Therefore, cardiorenal syndrome is a term that commonly refers to the collective dysfunction of the kidney and heart, resulting in cascades of feedback mechanisms with subsequent damage to both organs. Multiple mechanisms are involved in the pathophysiology of cardiorenal syndrome, whereby hemodynamic, neurohormonal, and oxidative stress play an important role, along with venous congestion and reduced arterial filling. Consecutive reduced renal perfusion and RAAS and sympathetic nervous system (SNS) activation contribute to oxidative stress and inflammation with subsequent acute and chronic kidney injury and heart failure [35].

Along with directly mediated cardiac disorders and MACEs, CKD displays a significantly elevated association with stroke risk, both ischemic and hemorrhagic [5,36,37,38]. Previous research revealed that stroke risk increased by 7% for every 10 mL/min/1.73 m^2^ decrease in eGFR and by 10% for every 25 mg/mmol increase in the urine albumin-to-creatinine ratio (UACR) [5,39] (Figure 1).

In the following sections, these relevant influencing factors and circumstances will be discussed.

## 3. Traditional Risk Factors and Their Impact on Cardiovascular Risk in CKD Patients

Traditional cardiovascular risk factors show a high prevalence in CKD patients, whereby their impact on arteriosclerotic vascular disease is clinically and prognostically relevant even in the early stages of CKD [24,40,41,42,43]. As an emerging global health burden, CKD is independently associated with elevated cardiovascular risk [40,41]. Along with macrovascular pathologies, traditional risk factors lead to the impairment of microvascular circulation, such as progredient nephrosclerosis in smaller kidney vessels, resulting in dramatically reduced life expectancy, with the loss of up to 25 years of life in advanced CKD stages compared to individuals with normal kidney function [35,44,45]. 

### 3.1. Arterial Hypertension

In this context, arterial hypertension plays an important role. On the one hand, arterial hypertension might be the consequence of worsening CKD development, and on the other hand, it might be the cause of progressing CKD [2,24]. Up to 90% of CKD patients are affected by arterial hypertension, whereby both are intimately related by an interplay of different factors, such as water and salt retention, consequent extracellular volume expansion, and endothelial dysfunction [46,47]. Activation of the SNS and upregulation of the RAAS play an important role in this pathophysiological interplay [46]. The subsequently high blood pressure variability in CKD patients requires close monitoring and appropriate monitoring strategies [2,46]. Patients’ individual blood pressure target values according to the latest guidelines and their potential adjustment during their lifetime individually due to the course of CKD development or pathophysiological remodeling need to be considered with highest priority [2]. Therefore, individual target values <130/80 mmHg and, if necessary, <120 mmHg systolic depending on underlying kidney disease and related comorbidities should be achieved through the combined use of the maximum tolerated dose of RAAS inhibition and diuretics and/or calcium channel blockers [2]. Therefore, current research such as the SPRINT trial suggests the use of lower blood pressure targets in selected groups of patients, while complementary KDIGO guidelines suggest lower individual blood pressure targets for CKD patients with significant proteinuria, deploying a cut-off of >300 mg/daily [47,48,49]. In daily clinical practice, challenges such as hyperkalemia or post-diuretic hypotension in CKD patients have to be faced while achieving individual blood pressure targets [47]. 

### 3.2. Smoking

Previous research revealed smoking as a traditional risk factor, as smoking negatively influences arteriosclerotic cerebrovascular and cardiovascular sequelae [40,43,50,51]. Smoking is negatively related to CKD progression and CKD-related CVD based on pathophysiological mechanisms such as endothelial function impairment or activation of the SNS by nicotine [2,52]. Therefore, lifestyle modifications in CKD populations including regular physical activity, appropriate weight loss, as well as smoking cessation seem to play an important role in positively influencing the further clinical course of CKD progression. Despite the absence of randomized trials, observational studies show significant clinical benefits after cessation [2,53]. 

### 3.3. Dyslipidemia

The lipoproteins high-density lipoprotein (HDL) and low-density lipoprotein (LDL) are established factors in CVD and MACE rates in CKD patients [2]. In accordance with the current guidelines of the European Society of Cardiology (ESC) from 2023, patients with CKD stage 3 are recommended to have an LDL-C target of 70 mg/dL (ApoB 80 mg/dL; non-HDL-C 100 mg/dL), while CKD patients in stage 4–5 are assigned to the high-risk cohort with an individual treatment goal for LDL-C below 55 mg/dL (ApoB 65 mg/dL; non-HDL-C 85 mg/dL) [54]. Based on CKD patients’ individual treatment goals, classical LDL lipid-lowering therapeutic strategies are based on oral medication, including statins, ezetimibe, and bempedoic acid, or the subcutaneous application of PCSK9 inhibitors [2,54,55]. Novel therapeutic approaches such as pelacarsen in the context of LDL and lipoprotein (a) cholesterol lowering reveal further therapeutic approaches in individualized lipid-lowering strategies in high-risk populations, such as CKD patients [56]. Future research needs to prove the potential beneficial effects of lipoprotein (a) lowering in renal disease, but currently, no scientific data or safety information in long-term studies is available [57]. 

Oral statin medication as a standard lipid-lowering therapy is linked to a significant MACE reduction of 22% per 40 mg/dL (1 mmol/L) LDL reduction (Cholesterol Treatment Trialists’ (CTT) Collaboration, 2010 [58]) and shows a logarithmic coherence between risk reduction in high-risk conditions, such as CVD, diabetes, and CKD, and LDL reduction [59]. These beneficial effects of orally administrated statin medication are diminished by worsening kidney function and cannot be proven for individuals with end-stage CKD 5d, as reported previously [2]. Known side effects such as myalgia, elevated transaminases, or other side effects occur more frequently in CKD and inevitably lead to an adjustment of the statin dose in the long term, which is more often used in lower doses [60]. The efficacy of oral statin therapy is diminishing in advanced CKD, as previous research on the AURORA study by Fellström et al. and Wanner et al. revealed [61,62]. Therefore, no significant effect of orally administrated statin therapy on composite MACE endpoints could be observed in patients with or without diabetes undergoing dialysis [61,62]. Thus, the clinical management of dyslipidemia, which is highly prevalent in CKD patients, is obviously challenging. The prevalence is estimated to be 45.5% in CKD stage 1 (eGFR > 90 mL/min/1.73 m^2^) and up to 67.8% in CKD stage 4 (eGFR 15–29 mL/min/1.73 m^2^) [63]. A sophisticated analysis of current evidence suggests the clinical use of statins, fibrates, or omega-3 fatty acid to be beneficial in CKD patients according to the individual cardiovascular risk. The use of ezetimibe, bempedoic acid, and PCSK9 inhibitors might be expanded in CKD patients as further scientific evidence is obtained [57,64].

### 3.4. Diabetes

A substantial proportion of diabetes patients will develop CKD during their lifetime due to the microvascular damage caused by diabetes, and therefore they display an increased risk for CVD and MACE rates [65]. While the global incidence of type 2 diabetes (T2D) is increasing continuously, CKD is estimated to affect 50% of T2D patients globally, and its negative impact on disease prognosis on diabetic kidney disease (DKD) has been revealed previously [66]. In daily clinical practice, the treatment of glycemic control in CKD patients is often challenging due to the altered kinetics of glucose-lowering medications in patients’ blood and variable blood glucose homeostasis, resulting in dysglycemia [67]. Previous epidemiological data as well as the KDIGO recommendations in 2022 on optimal glycemic control in CKD patients revealed an HbA1c range between 6 and 8%, as well as between 7 and 9%, to be favorable to preventing hypoglycemia in multimorbid patients, being associated with better clinical outcomes in CKD patients [2,67]. The Action to Control Cardiovascular Risk in Diabetes (ACCORD) study revealed in patients with impaired kidney function a significant higher cardiovascular and all-cause mortality when subjected to strictly glycemic control (HbA1c level 6.0%) compared to the standard control (HbA1c level 7.0–7.9%) [68]. In younger patients without major comorbidities, better glycemic control with individual HbA1c levels <6.5% might be achieved [51]. Along with individual lifestyle modifications, the potential association between antidiabetic medication given to T2D patients and CKD is firstly based on the oral intake of metformin and sodium glucose cotransporter 2 inhibitors (SGLT2 inhibitors), whereby the individual adjustment of glucose-lowering medication due to impaired renal function must always be considered and re-evaluated [2,51]. In this context, individual medication adjustment, especially while administrating metformin or sulfonylureas, plays an important role in order to prevent medication-associated hypoglycemia [69].

Along with their glucose-lowering effect, various beneficial pleiotropic effects in SGLT2 inhibitors have been demonstrated in previous trials, i.e., the EMPA-Reg outcome, CREDENCE, and DAPA-CKD [70,71,72,73]. Therefore, SGLT2 inhibitors may exhibit anti-inflammatory properties and have been proven to induce significant improvements in cardiovascular and renal outcomes due to their improvement of endothelial function and subsequent vasodilation, as well as preserving cardiac contractility via their anti-fibrotic and cardioprotective effects [74]. Additionally, glucagon-like peptide-1 receptor agonists (GLP1-RAs) are known for their MACE reduction in T2D patients. Their potential beneficial effects on kidney outcome are currently being explored by focusing on the effects of GLP1-RAs on kidney inflammation and perfusion, whereby positive protective effects on natriuresis, diuresis, and oxidative stress might be assumed [75]. 

## 4. Pathophysiological Mechanisms Contributing to Increased Cardiovascular Risk in CKD

Patients with CKD are exposed to a significantly increased cardiovascular risk that cannot be fully explained by traditional cardiovascular risk factors alone. In addition to hypertension, diabetes, dyslipidemia, and smoking, CKD triggers a range of non-traditional, disease-specific mechanisms that promote vascular and cardiac damage [1].

**Figure 2:**
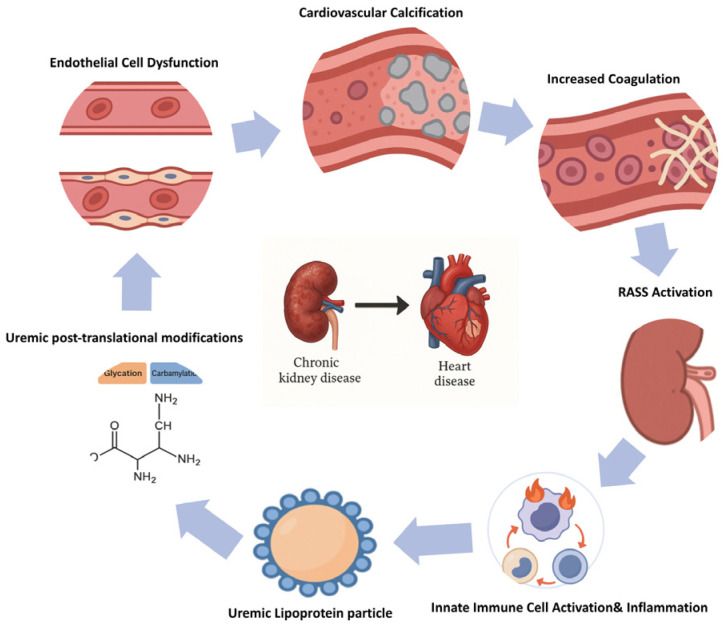
Pathophysiological mechanisms contributing to increased cardiovascular risk in CKD.

These mechanisms include the accumulation of protein-bound uremic toxins, post-translational protein and lipoprotein modifications, chronic low-grade inflammation, endothelial dysfunction, hypercoagulability, and vascular calcification, as well as the maladaptive activation of neurohormonal systems such as the RAAS and the SNS. Furthermore, CKD-associated anemia contributes to myocardial stress and adverse cardiac remodeling (Figure 2).

In the following section, we describe eight interrelated pathophysiological mechanisms that underlie the increased cardiovascular morbidity and mortality in CKD. For each pathway, we summarize relevant molecular mediators, describe their impact on cardiovascular function and structure, and—where applicable—discuss current or potential therapeutic approaches. Finally, we highlight how these mechanisms interact and collectively shape the cardiovascular phenotype in patients with CKD [24].

### 4.1. Uremic Toxins

Progressive renal impairment leads to the retention of more than 140 uremic solutes, as cataloged by the EUTox Work Group (EUTox Database, accessed 2025, [76]). Among these, protein-bound toxins such as indoxyl sulfate (IS) and p-cresyl sulfate (PCS) play a particularly deleterious role in cardiovascular pathology. These toxins originate from the microbial metabolism of dietary tryptophan and tyrosine in the gut and accumulate in patients with reduced renal clearance [22].

Mechanistically, IS and PCS activate the aryl hydrocarbon receptor (AhR) pathway in endothelial and vascular smooth muscle cells, leading to the increased generation of reactive oxygen species (ROS), the upregulation of pro-inflammatory cytokines via NF-κB activation, and the suppression of protective antioxidant pathways such as Nrf2 [13,15]. In human endothelial cell cultures and in vivo CKD models, IS exposure resulted in the increased expression of adhesion molecules (VCAM-1, ICAM-1), impaired nitric oxide synthesis, and accelerated vascular calcification [12,34].

Importantly, asymmetric dimethylarginine (ADMA), a uremic toxin that acts as an endogenous nitric oxide synthase inhibitor, independently predicts cardiovascular mortality in CKD [77]. Similarly, trimethylamine-N-oxide (TMAO) and symmetric dimethylarginine (SDMA)—gut-derived metabolites—have been implicated in vascular inflammation and atherogenesis [12].

The gut microbiota is now recognized as a central source of several of these uremic toxins, highlighting the concept of a gut–kidney–heart axis. In CKD, altered microbial composition (“dysbiosis”) and disruption of the intestinal epithelial barrier promote systemic absorption of harmful metabolites, including IS and TMAO [34]. Interventions targeting the microbiota, such as prebiotics, synbiotics, and low-protein diets with keto-analogs, have shown reductions in toxin levels in small trials, although long-term cardiovascular outcome data are still lacking [25].

Therapeutically, the oral charcoal adsorbent AST-120 has been shown to lower plasma levels of IS and PCS in multiple clinical studies, although its effect on hard cardiovascular endpoints remains controversial [13]. Innovative extracorporeal removal strategies using sorbent-enhanced dialysis membranes or adsorptive columns are under development, aiming to improve the clearance of protein-bound toxins beyond that achievable with conventional dialysis [22]. 

Overall, the retention of uremic toxins in CKD promotes cardiovascular risk through endothelial dysfunction, oxidative stress, and inflammation. While the mechanistic understanding is growing, interventional strategies remain limited, and further studies are required to evaluate their impact on cardiovascular outcomes.

### 4.2. Uremic Post-Translational Modifications and Uremic Lipoprotein Particles

The uremic milieu promotes several pathological post-translational modifications (PTMs), including carbamylation, guanidinylation, oxidation, and glycation. These alterations affect structural and functional proteins, lipoproteins, and enzymes, contributing to vascular and cardiac injury [15,21].

Carbamylation of circulating proteins occurs through isocyanic acid, derived from urea or myeloperoxidase activity, and has been associated with accelerated vascular calcification, particularly in coronary arteries. Notably, carbamylated sortilin induces osteogenic transformation in vascular smooth muscle cells and correlates with the coronary calcium burden in CKD [24]. 

Guanidinylated apolipoprotein C-III (ApoC3) triggers pro-inflammatory signaling in monocytes and promotes endothelial dysfunction, directly linking modified lipoproteins to vascular inflammation [21]. Similarly, advanced glycation end-products (AGEs) accumulate independently of hyperglycemia and promote endothelial activation through receptor for AGE (RAGE) signaling, contributing to arterial stiffening and atherogenesis [16].

In addition, advanced oxidation protein products (AOPPs), particularly oxidized albumin, impair endothelial nitric oxide availability, enhance oxidative stress, and are independently associated with cardiovascular mortality in dialysis patients [78].

Uremia also leads to structural changes in lipoprotein particles. LDL cholesterol is enriched in carbamylated and oxidized forms, which enhances its atherogenic potential. Conversely, HDL particles lose their anti-inflammatory and antioxidant capacity due to the incorporation of uremic toxins, structural distortion, and altered enzyme interactions [2,9,16]. The net result is a dysfunctional HDL profile with impaired reverse cholesterol transport and endothelial protection.

While these molecular alterations are well-documented, therapeutic interventions remain limited. Some agents such as pyridoxamine and AGE crosslink breakers have been explored in preclinical models but have not yet reached routine clinical use. In the dialysis setting, high-flux membranes and adsorptive dialyzers may modestly reduce circulating AOPPs and carbamylated proteins, but outcome data are scarce. Targeting uremic dyslipidemia with conventional lipid-lowering therapy remains a cornerstone but is often insufficient to reverse the underlying lipoprotein dysfunction in advanced CKD.

### 4.3. Innate Immune Activation, Inflammation, and Oxidative Stress

CKD is characterized by a sustained state of low-grade inflammation and oxidative stress, driven by the interplay of uremic toxins, dysbiosis, and comorbid conditions such as diabetes and hypertension [15]. 

Activation of innate immunity occurs early, with elevated levels of pro-inflammatory cytokines such as IL-1β, IL-6, and TNF-α, many of which are regulated by the NF-κB pathway. In CKD, monocyte activation and macrophage infiltration are prominent, particularly in perivascular and cardiac tissue [15,78]. 

Recent evidence highlights the role of the NLRP3 inflammasome, a cytosolic multiprotein complex activated by mitochondrial dysfunction, uremic toxins, and uric acid crystals. Its activation promotes the release of IL-1β and IL-18 and contributes to vascular remodeling and myocardial fibrosis in CKD [21]. 

Oxidative stress is exacerbated by an imbalance between reactive oxygen species (ROS) and antioxidant defense mechanisms. Uremic toxins such as indoxyl sulfate impair Nrf2 signaling, a key transcription factor regulating antioxidant responses, thereby perpetuating cellular injury [13,15]. 

Emerging concepts such as gut–kidney axis dysbiosis further link systemic inflammation to intestinal barrier dysfunction and endotoxemia. Translocation of lipopolysaccharides (LPS) from the gut into the circulation activates Toll-like receptor 4 (TLR4), amplifying systemic inflammation and vascular damage [34].

Clinically, elevated IL-6 and TNF-α levels correlate with left ventricular hypertrophy, arterial stiffness, and increased cardiovascular mortality in CKD [16]. 

However, interventional trials targeting inflammation remain scarce. Canakinumab, an IL-1β antibody, resulted in reduced MACE incidence in the general population [79], but its use in CKD remains exploratory. Ziltivekimab, an IL-6-targeting antibody, is currently under investigation in the CKD population [80]. 

Antioxidant therapies such as N-acetylcysteine, bardoxolone methyl, or dietary polyphenols have shown mixed results. Current guidelines do not recommend their routine use due to limited outcome data [13]. More targeted anti-inflammatory strategies tailored to CKD-specific mechanisms may represent a promising future direction.

### 4.4. Endothelial Dysfunction

Endothelial dysfunction is a hallmark of cardiovascular disease in CKD and precedes overt atherosclerosis. In uremia, the endothelium shifts toward a pro-inflammatory, pro-thrombotic, and vasoconstrictive phenotype [15,16]. 

Key molecular mechanisms include reduced nitric oxide (NO) bioavailability, due to the impaired activity of endothelial nitric oxide synthase (eNOS) and increased production of asymmetric dimethylarginine (ADMA), an endogenous NO synthase inhibitor [81]. Elevated levels of endothelin-1 (ET-1) further promote vasoconstriction and oxidative stress.

CKD-related inflammation increases the expression of adhesion molecules such as VCAM-1, ICAM-1, and E-selectin, which mediate leukocyte adhesion and transmigration across the endothelium. This process fosters vascular inflammation and remodeling [82]. 

In addition, the activation of NF-κB signaling pathways in endothelial cells upregulates cytokine production and perpetuates the inflammatory milieu. Uremic toxins such as indoxyl sulfate and p-cresyl sulfate trigger oxidative stress, mitochondrial dysfunction, and endothelial senescence [13].

The endothelium in CKD also contributes to procoagulant activity by increasing tissue factor expression and reducing thrombomodulin, favoring thrombin generation and fibrin deposition. This promotes both microvascular and macrovascular thrombotic risk [83].

Importantly, endothelial dysfunction is not only a precursor to atherosclerosis but also directly contributes to arterial stiffness, left ventricular hypertrophy, and impaired myocardial perfusion. It serves as a key mediator in the development of both atherosclerotic and non-atherosclerotic cardiovascular events in CKD [84].

While lifestyle modification and treatment of classical risk factors remain fundamental, no specific endothelial-targeted therapy is currently approved. Experimental agents such as ET-1 receptor antagonists, Nrf2 activators, and selective anti-oxidative therapies are under investigation but are not yet part of routine care.

### 4.5. Hypercoagulability and Altered Platelet Function

Patients with CKD exhibit a complex hemostatic imbalance characterized by both prothrombotic and bleeding tendencies. Despite a higher risk of hemorrhage, especially in advanced CKD, a procoagulant state is increasingly recognized as a major contributor to cardiovascular events, including stroke, venous thromboembolism, and vascular access thrombosis [83]. 

Endothelial dysfunction promotes tissue factor expression, reduces thrombomodulin, and increases the circulating levels of von Willebrand factor, collectively favoring thrombin generation and fibrin deposition. This contributes to a hypercoagulable vascular environment in CKD [15,82]. 

Simultaneously, platelet activation is enhanced via increased expression of P-selectin, altered serotonin storage, and impaired nitric oxide signaling. These changes contribute to platelet aggregation in dialysis patients [85]. 

Inflammation and oxidative stress further drive this process through the upregulation of procoagulant cytokines (e.g., IL-6 and TNF-α) and the increased generation of reactive oxygen species, which impair endothelial antithrombotic function [15,86]. 

Anticoagulant therapy in CKD remains challenging due to altered pharmacokinetics and bleeding risk. Vitamin K antagonists may increase vascular calcification, whereas DOACs require cautious dose adjustment. Ongoing trials are investigating novel anticoagulants with renal-safe profiles, including factor XI inhibitors.

### 4.6. Cardiovascular Calcification

Cardiovascular calcification is a highly prevalent complication in CKD and contributes significantly to increased arterial stiffness, left ventricular hypertrophy, and cardiovascular mortality. Unlike atherosclerosis-related intimal calcification in the general population, CKD patients often develop medial vascular calcification (Monckeberg sclerosis), which is not necessarily lipid-driven [84,87].

The pathogenesis of vascular calcification involves an active, cell-regulated process resembling bone formation. Vascular smooth muscle cells (VSMCs) undergo osteogenic transformation under the influence of hyperphosphatemia, hypercalcemia, and chronic inflammation. Key mediators include bone morphogenetic proteins (BMPs), Runx2, and alkaline phosphatase [88].

Hyperphosphatemia induces phosphate uptake into VSMCs via PiT-1 transporters, triggering the expression of osteochondrogenic genes. Inflammation and oxidative stress amplify this transformation and promote matrix vesicle release, which initiates extracellular matrix mineralization [89].

Vitamin K deficiency, often observed in CKD, leads to reduced activation of matrix Gla protein (MGP), a potent inhibitor of vascular calcification. Similarly, the accumulation of uremic toxins and the disruption of calcium–phosphate homeostasis disrupt natural anti-calcification mechanisms [90]. 

Cardiovascular calcification in CKD has been independently associated with myocardial ischemia, heart failure with a preserved ejection fraction (HFpEF), and sudden cardiac death. Its presence is a strong predictor of adverse cardiovascular outcomes across all stages of CKD, including patients on dialysis [91]. 

Despite its clinical relevance, no specific therapy has proven efficacy in reversing vascular calcification. Phosphate binders, vitamin D analogs, and calcimimetics are commonly used to mitigate mineral imbalance but have inconsistent effects on calcification progression. Emerging strategies include vitamin K supplementation, SNF472, and bisphosphonates, though these remain experimental.

### 4.7. RAAS and SNS Activation

Dysregulation of the RAAS and SNS plays a central role in the progression of CKD and the development of cardiovascular complications.

In CKD, RAAS is persistently activated, leading to increased levels of angiotensin II and aldosterone. These hormones promote vasoconstriction, sodium retention, fibrosis, and inflammation, thereby contributing to hypertension, vascular remodeling, and left ventricular hypertrophy [92]. 

Angiotensin II further activates NADPH oxidases, increasing reactive oxygen species (ROS) production, and upregulates pro-inflammatory cytokines such as IL-6 and TGF-β. This links RAAS activation to both oxidative stress and immune dysregulation in CKD [15].

Aldosterone, in turn, induces endothelial dysfunction and vascular calcification and has direct profibrotic effects on the myocardium and kidneys. It also promotes mineralocorticoid receptor (MR)-mediated inflammation, which is particularly relevant in DKD [93].

The SNS is similarly overactive in CKD due to afferent renal signaling and impaired baroreceptor function. This enhances heart rate, vascular resistance, and RAAS activity, generating a self-reinforcing loop. Elevated norepinephrine levels correlate with increased mortality in CKD patients [94]. 

Novel concepts such as organ-specific RAAS and the “crosstalk model” describe the dynamic interplay between cardiac and renal RAAS activity. In early CKD, systemic inhibition may suffice, whereas advanced stages may require combined or local interventions [95].

Therapeutically, RAAS blockade remains a cornerstone of cardiovascular risk reduction. ACE inhibitors, ARBs, and mineralocorticoid receptor antagonists (MRAs) like finerenone have demonstrated benefits in CKD, especially in diabetic cohorts [93].

Additionally, renal denervation, baroreceptor activation therapy, and biofeedback-guided autonomic modulation represent emerging SNS-targeting interventions, although their long-term efficacy and safety in CKD remain under investigation.

### 4.8. Anemia

Anemia is a common and multifactorial complication of CKD that significantly contributes to cardiovascular morbidity, fatigue, and reduced quality of life. Its pathophysiology extends beyond reduced erythropoietin (EPO) synthesis and includes chronic inflammation, iron dysregulation, and endothelial dysfunction [10]. 

Inflammatory cytokines, particularly IL-6, stimulate hepatic production of hepcidin, which inhibits intestinal iron absorption and macrophage iron release. This functional iron deficiency limits erythropoiesis even when iron stores are adequate. Concurrent oxidative stress impairs EPO receptor signaling and red blood cell survival [96].

Anemia in CKD also contributes to endothelial dysfunction, increases myocardial oxygen demand, and exacerbates left ventricular hypertrophy, forming a vicious cycle with cardiovascular disease.

The mainstay of therapy consists of erythropoiesis-stimulating agents (ESAs) and iron supplementation, administered either orally or intravenously. However, ESA therapy carries risks, including hypertension, thromboembolic events, and possible tumor progression in cancer patients. Targeting hemoglobin levels >13 g/dL has been associated with increased cardiovascular events [97,98].

Recently, hypoxia-inducible factor prolyl hydroxylase inhibitors (HIF-PHIs) have emerged as a promising alternative. These agents stimulate endogenous EPO production and promote iron mobilization by reducing hepcidin levels. Preliminary studies suggest a lower risk of hypertension and improved lipid profiles compared to ESAs [99,100]. 

Clinical guidelines (e.g., KDIGO 2021) recommend initiating anemia work-up in CKD patients with Hb < 12 g/dL and considering ESA therapy when Hb is <10 g/dL, after correcting iron deficiency. Ferritin and transferrin saturation (TSAT) remain standard biomarkers to guide iron management [20].

Further long-term studies are needed to assess the safety and cardiovascular efficacy of HIF-PHIs, particularly in dialysis-dependent and high-risk CKD populations.

In summary, a broad array of interrelated pathophysiological mechanisms contributes to the elevated cardiovascular morbidity observed in patients with chronic kidney disease. Chronic inflammation, endothelial dysfunction, oxidative stress, coagulation abnormalities, neurohormonal activation, and anemia interact in a complex manner, resulting in vascular remodeling and myocardial dysfunction. Improved understanding of these interconnected pathways not only enhances risk stratification but also opens the door to targeted therapeutic strategies, particularly those focused on anti-inflammatory, endothelial-protective, and cardiorenal-modulating interventions.

## 5. Cardiovascular Biomarkers and Their Prognostic Impact on Cardiovascular Risk Assessment

Progressive heart failure and negative CVD outcomes such as ACS—based on progressive CAD—remain an ongoing threat for CKD patients [101]. Referring to this background, various cardiovascular biomarkers have been established and studied for early detection, monitoring, and prognosis estimation in CKD, acknowledging that these biomarkers might be easily affected by impaired kidney function and therefore their clinical use and meaningfulness might be limited in CKD due to eGFR-dependent thresholds [101]. Nevertheless, the clinical application of cardiovascular biomarkers in CKD patients is widespread, with the following parameters playing the most important role in clinical practice: those associated with myocardial stretch, i.e., N-terminal pro-BNP (NT-proBNP), and those displaying myocardial damage, i.e., high sensitivity troponin T (hs TnT). Cystatin C (Cys C), heart-type fatty acid-binding protein (H-FABP), soluble growth stimulating gene 2 (sST2), galectin-3 (Gal-3), growth differentiation factor-15 (GDF-15), neutrophil gelatinase-associated lipocalin (NGAL), and kidney injury molecule-1 (KIM-1) play a minor role in everyday clinical practice and are not determined as standard [101]. Additionally, the latest research data on the prognostic clinical use of novel biomarkers, such as fibroblast growth factor 23 (FGF-23), which is expressed by cardiomyocytes and enhances RAAS activation, as well as copeptin—the C-terminal fragment of provasopressin—and the mid-regional pro adrenomedullin (MR-proADM) emphasize their emerging role in short-term risk stratification and providing additional information to the established biomarkers [102]. Vascular calcification represents a relevant phenomenon in CKD patients and patients with end-stage renal disease. Therefore, discovered about 20 years ago, microRNAs have been reported to play an essential role in gene expression and protein synthesis. Many microRNAs are involved in vascular calcification, whereby decreased circulating levels of several microRNAs involved in the vascular smooth muscle cell (VSMC) phenotype, including miR-125b, miR-155 and miR-145, have been reported in patients with CKD stage 3 and 4. Particular emphasis is placed on endothelial-specific miR-126, a potential biomarker of endothelial dysfunction [103]. Therefore, the abnormalities in microRNA play an important role in initializing and promoting the calcification pathway in CKD patients [103,104]. The potential utility of microRNA—both as a diagnostic and a prognostic biomarker—in CKD has been advocated, whereby its role in CKD pathophysiology and its expression pattern has to be further verified for a potential clinical application in the future [105]. Regarding these new biomarkers, most of them lack large cohort studies proving their diagnostic and prognostic value due to their limited clinical application [102].

In this context, DeFilippi et al. revealed in their previous research on NT-pro BNP assessment in renal disease no further adjustment for renal function and confirmed the recommended cut points of 450, 900 and 1800 ng/L for those aged <50, 50–75, and >75 years [106]. Nevertheless, fibrosis, matrix remodeling, and inflammation play a key role in heart failure and CKD and therefore may affect myocardial cells, reducing the diagnostic value of cardiac biomarkers [101]. In conclusion, the classical cardiovascular biomarkers, i.e., NT-proBNP and hs TnT, certainly have clinical significance, with both advantages and limitations [101,105].

### 5.1. N-Terminal Pro-BNP (NT-ProBNP)

In the pathophysiological context of CKD and heart failure, cardiac preload is often negatively influenced by fluid retention and sodium accumulation and furthermore results in increased wall pressure and subsequent NT-proBNP release [101]. NT-proBNP secretion from cardiac myocytes is mediated by various stimuli, i.e., pressure and volume overload [77,107] and left ventricular hypertrophia, as well as endothelial dysfunction, inflammation, and reduced renal clearance in CKD [108,109]. Previous research in patients with CKD indicated an association between adverse kidney outcome and cardiac biomarkers. In this context, among participants with CKD and T2D in the Reduce Cardiovascular Events with Aranesp Therapy (TREAT) trial, elevated NT-proBNP and elevated troponin values were associated with a higher risk of end-stage renal disease [110]. Bansal et al. reported in a community-based, multicenter cohort of older adults free of clinical heart failure a significantly higher loss of rapid kidney function decline for the highest levels of NT-proBNP and troponin levels [77]. These associations between cardiac biomarkers and rapid kidney function decline seem to be independent of other influencing factors, such as other CVD risk factors or the baseline level of eGFR [77]. Therefore, elevated NT-proBNP levels in clinically asymptomatic patients seem to indicate an association between biomarker level and potential kidney function decline and subclinical CVD risk [111,112]. Even the pre-cardiac surgery assessment of NT-proBNP levels revealed an up to three-fold higher risk of postoperative acute kidney injury provided by preoperative elevated NT-proBNP levels [113].

These results indicate that patients with elevated NT-proBNP levels and even subclinical cardiovascular disease are at high risk for developing adverse kidney outcomes and incident CKD [77]. 

### 5.2. High-Sensitivity Troponin T (Hs TnT)

Along with the previously mentioned negative association between elevated NT-proBNP levels and poor cardiovascular outcome, elevated hs TnT levels predict negative CVD association in CKD [77]. Therefore, Bansal et al. revealed a negative association between the highest hs TNT levels—in comparison to undetectable hs TnT—and a high risk of rapid kidney decline [77]. These association seem to be independent of other preconditions, such as prevalent CKD at baseline, sex, age or baseline eGFR [77]. The previously mentioned TREAT trial elucidated in participants with T2D and CKD a significant higher progression rate to end-stage renal disease for those with detectable hs TnT measurements in this cohort [110].

These results suggest that subclinical CVD, potentially characterized by elevated hs TnT levels, may be a noticeable clinical contributor to the rapid decline of kidney function in the elderly [77]. Nevertheless, previous trials revealed among CKD patients with no-self reported history of CVD elevated levels of NT-proBNP and hs TnT above the conventional upper reference limits (URLs) for 40% to 43% of participants [114]. In end-stage kidney disease patients with eGFR < 30 mL/min/1.73 m^2^, 68% of participants had concentrations of hs TnT above the conventional URLs [114]. Along with the elevated biomarker levels in CKD due to various preconditions, CKD patients are estimated to be at high risk for CVD and MACEs. Therefore, symptoms concerning ACS or CAD and elevated hs TnT levels are challenging in daily clinical practice and require an educated and nuanced approach [115,116,117]. In this context, a systematic review of 23 studies of patients with CKD demonstrated that the sensitivity of hs TnT assessment for ACS diagnosis ranged from 71 to 100% and the specificity ranged from 31 to 86% when applying the reference values used in the general population [114].

In summary, since the diagnostic and prognostic utility of cardiac biomarker assessment is limited due to a single point value determination, cardiac biomarker determination should be combined and monitored at multiple time points to achieve optimal clinical prediction [101]. Future efforts to develop individualized upper reference limits for these commonly used cardiac biomarkers are urgently needed to specify eGFR-dependent thresholds for an operationalized daily clinical approach [114]. Although these cardiac biomarkers contribute to the early detection of subclinical CVD and play an important role in the clinical follow-up and monitoring in CKD patients, there remains predictive and diagnostic uncertainty. Future prospective interventional data are urgently needed to verify the utility and clinical role of these cardiac biomarkers in CKD patients.

## 6. Conclusions

This review highlights the importance of classic cardiovascular risk factors’ assessment, the understanding of specific CKD-associated pathophysiological alterations, and the clinical use of cardiac biomarkers with critical values adjusted to eGRF in individualizing CKD patients’ therapeutic strategies and to minimize the burden of CKD.

Since patients with CKD are at very high risk of developing CVD and MACEs during their lifetime, the optimal adjustment of traditional cardiovascular risk factors and understanding CKD-specific pathophysiological mechanisms seem to be of great importance during CKD patients’ medical care. While the definition and assessment criteria for MACEs vary across previous studies, a critical interpretation is needed to compare and evaluate previous scientific evidence in CKD patients.

Unraveling the complex and multifactorial interplay of different risk factors and pathophysiological mechanisms in CKD population will contribute to optimized and individualized therapeutic approaches including novel strategies targeting chronic low-grade inflammation in CKD patients. The previously published data from the CANTOS trial (canakinumab) or RESCUE trial (ziltivekimab) revealed interesting aspects regarding this approach [79,80]. Further development and optimization of individualized therapeutic strategies in CKD patients with concomitant high risk for CVD and MACEs should be achieved to improve CKD patient prognosis and minimize CKD patient morbidity and mortality. Therefore, future studies focusing on the assessment of targeted therapies, such as anti-inflammatory approaches, are urgently needed to demonstrate whether suppressing inflammation definitively reduces cardiovascular risk and improves cardiovascular outcomes in CKD.

Educated and nuanced approaches to cardiac biomarkers, even in CKD patients with subclinical cardiac disease, might support and improve individual risk stratification. Therefore, further scientific medical efforts are urgently needed to develop eGFR-specific thresholds for commonly used cardiac biomarkers in the setting of CKD to improve their predictive significance in heart failure and CVD risk assessment [114].

Despite all the medical advancements made through personalized medicine, specific anti-inflammatory approaches, and the integrated use of adapted established biomarkers and potential novel biomarkers, real-world barriers remain in everyday clinical practice in CKD patients. An individual CKD patient’s medical care is likely to be shaped by obstacles such as high costs for effective new targeted therapies, insurance coverage, cost-effectiveness, and resource limitations. Targeted anti-inflammatory therapies and novel biomarkers remain prohibitively expensive and might not be universally accessible to all CKD patients.

Further scientific effort and additional trials are needed to determine the clinical benefit for CKD patients by treating early stages of cardiac dysfunction to minimize the burden and progression of kidney disease.

## Data Availability

Not applicable.
